# The* CD40* rs1883832 Polymorphism Affects Sepsis Susceptibility and sCD40L Levels

**DOI:** 10.1155/2018/7497314

**Published:** 2018-03-26

**Authors:** Zuo-Liang Liu, Jing Hu, Xue-Fei Xiao, Yue Peng, Shang-Ping Zhao, Xian-Zhong Xiao, Ming-Shi Yang

**Affiliations:** ^1^Translational Medicine Center of Sepsis, Department of Pathophysiology, The Third Xiangya Hospital, Central South University, Changsha, Hunan, China; ^2^Department of Critical Care Medicine, The Third Xiangya Hospital, Central South University, Changsha, Hunan, China; ^3^Department of Pathophysiology, Xiangya School of Medicine, Central South University, Changsha, Hunan, China

## Abstract

Sepsis is a severe and progressive disease characterized by systemic inflammatory response syndrome (SIRS). CD40 serves as a vital link between immune response and inflammation. This study was designed to investigate the potential association between a functional single-nucleotide polymorphism (SNP) of* CD40* (rs1883832) and susceptibility to sepsis. We first performed a case-control study to explore the relationship between the* CD40* rs1883832 polymorphism and sepsis.* CD40* mRNA expression and protein expression were determined by real-time PCR and western blotting, respectively, in peripheral blood mononuclear cells (PBMCs) from sepsis patients and healthy controls. The plasma sCD40L levels in the two groups were measured by ELISA. The results showed that the frequencies of the TT genotype and the* CD40* rs1883832 T allele were significantly higher in sepsis patients than in healthy controls. Plasma sCD40L levels were also significantly increased in sepsis patients. In addition, TT genotype carriers among sepsis patients displayed the highest* CD40* expression at both the mRNA and protein levels, accompanied by the highest plasma sCD40L concentrations. In conclusion, the* CD40* rs1883832 T allele acts as a risk factor for increased susceptibility to sepsis and may be involved in the process of sepsis through regulation of* CD40* expression and plasma sCD40L levels.

## 1. Introduction

Sepsis is a disease caused by systemic or local infection and characterized by systemic inflammatory response syndrome (SIRS) [1–3]. Sepsis can lead to shock, multiple organ failure, and even death, especially if appropriate treatment is not implemented in time [4, 5]. The identification of an effective method for predicting the incidence rate of sepsis may result in the development of new strategies for the prevention and treatment of sepsis.

Sepsis patients in different stages of the disease will exhibit different immune statuses [6]. In the early stage of sepsis, the complement system, immune cells, and vascular epithelial cells will be activated by injury, foreign matter, or multiple infection factors, so the local and systemic defense response will also be activated. The immune systems of sepsis patients may become suppressed in the later stage of sepsis, showing decreased lymphocyte proliferation ability [7, 8]. The different immune statuses in sepsis mainly depend on the balance between proinflammatory cytokines and anti-inflammatory cytokines. Type II cytokines play a crucial role in sepsis which include the tumor necrosis factor (TNF) family, the interferon (IFN) family, and the immunoglobulin family. CD40 belongs to the TNF family of type II cytokines and is expressed on various cell types, including immunity cells, platelets, fibroblasts, epithelia cells, endothelial cells (ECs), smooth muscle cells (SMCs), mast cells, and dendritic cells (DCs) [9]. It is generally accepted that CD40 participates in local and systemic inflammatory responses, mediates immune cell proliferation and differentiation, and plays a crucial role in the regulation of the immune system.

Emerging studies have focused on the effects of CD40 and its ligand CD40L on the process of sepsis. In a lethal polymicrobial sepsis mouse model,* CD40* (−/−) mice exhibited significant improvement in mortality, lung injury, and inflammatory cytokine production compared with control mice. A further study in sepsis patients showed increased monocyte expression of CD40 compared with healthy control subjects [10]. Another study suggested that anti-CD40 treatment increased the level of antiapoptotic protein Bcl-x(L) in splenic B and T cells as well as in thymic T cells, provided nearly complete protection against sepsis-induced lymphocyte apoptosis, and improved the prognosis of sepsis [11]. An abnormal elevation of sCD40L was also observed in sepsis-associated encephalopathy and abdominal sepsis. By investigating the underlying mechanisms, the researchers found that Rac1 controlled surface mobilization of CD40L on activated platelets, so targeting Rac1 signaling might be a useful way to control the increased level of sCD40L in sepsis [12–14]. But there are also some recent studies that challenge the role of CD40/CD40L in sepsis and inflammatory disorders. For example, Michels and his colleagues did not find an association of anti-CD40 treatment with better survival rates in a sepsis-associated encephalopathy rat model [15]. Other scientists have also pointed out that the levels of sCD40L are not solely related to the presence and severity of sepsis [16]. Furthermore, limited studies have reported the possible association between* CD40* gene polymorphisms and sepsis susceptibility, though relevant polymorphisms have been identified to play an essential role in atherosclerosis, ischemic stroke, and Kawasaki disease [17–20].

In this study, we explored the association between a functional single-nucleotide polymorphism (SNP) in the* CD40* Kozak (rs1883832) sequence and susceptibility to sepsis. We also explored the association of this* CD40* polymorphism with CD40 expression in peripheral blood mononuclear cells (PBMCs) from sepsis patients and healthy controls, and the plasma sCD40L concentration was also examined to determine its role in sepsis.

## 2. Materials and Methods

### 2.1. Subjects

Our study cohort consisted of 583 Chinese Han subjects, with 261 patients diagnosed with sepsis and 322 matched healthy controls in the Third Xiangya Hospital between July 2013 and September 2015. All sepsis patients were enrolled from the ICU and healthy controls were enrolled from the Health Management Center. Sepsis was defined according to the standard criteria [21]. Five-milliliter venous blood samples were drawn into ethylene-diamine-tetraacetic acid (EDTA) containing tubes from all subjects for genotyping and blood biochemistry tests. Plasma and peripheral leukocytes were isolated immediately and stored separately at −20°C until analysis. Patient exclusion criteria were a history of malignant tumors, human immunodeficiency virus, autoimmune diseases, blood diseases, ACI, or other organic diseases. No significant differences were observed in age or gender between sepsis patients and healthy controls (Table 1).

Written informed consent was obtained from all subjects. The study was performed with the approval of the Ethical Committee of the Third Xiangya Hospital of Central South University.

### 2.2. Genotyping

Genomic DNA was purified from the whole blood samples with the standard phenol/chloroform protocol and stored at −20°C. A polymerase chain reaction (PCR) test was performed on a personal thermal cycler (Biometra®, Germany). To genotype the rs1883832 polymorphism, the following primers were produced according to a previous report [18]: 5′-ACCATGCCTCCTCCCGTAC-3′ (sense); 5′-CCACTCCCAACTCCCGTC T-3′ (antisense). A 20 *μ*L PCR reaction was performed containing 10 *μ*L 2x PCR buffer, 1 *μ*L of each primer (10 pmol), 2 *μ*l genomic DNA template, and 6 *μ*L ddH_2_O. The PCR amplification conditions were a denaturation step at 95°C for 5 min, followed by 35 cycles of denaturation at 94°C for 30 s, annealing at 60°C for 30 s, and extension at 72°C for 30 s, with a final incubation at 72°C for 10 min. Genotype analysis was performed through DNA sequencing completed by Sangon Biological Company.

### 2.3. Determination of sCD40L Levels

Plasma levels of sCD40L were measured by using an ELISA assay kit (Quantikine CD40 Ligand, R&D Systems) according to the manufacturer's instructions.

### 2.4. Isolation of PBMCs from Sepsis Patients and Controls

PBMCs were collected from 2 times diluted blood sample after centrifugation at 2000*g* for 30 min upon 5 ml Ficoll-Hypaque gradients (Sigma, USA). The PBMCs obtained were washed twice with ice-cold PBS and suspended in RPMI 1640 containing 10% fetal calf serum (FCS). Then, PBMCs were cultured in a 5% CO_2_ incubator at 37°C.

### 2.5. Real-Time PCR Analysis

Total RNA was extracted from PBMCs using TRIzol reagent (Qiagen) according to the manufacturer's instructions. cDNA was prepared using PrimeScript 1st Strand cDNA Synthesis Kit (TaKaRa, Japan). Real-time PCR was performed using the real-time PCR Master Mix (ToYoBo, Japan) with ABI 7300 real-time PCR system according to the manufacturer's protocol. Sequences of* CD40* primers were as follows: 5′-GCAGGCACAAACAAGACTGA-3′ (sense) and 5′-TCGTCGGGAAATT GATCTC-3′ (antisense); sequences of *β*-actin primers (endogenous control) were: 5′-ACTCTTCCAGCCTTCCTTCC-3′ (sense); and 5′-CGTACAGGTCTTTGCGGATG-3′ (antisense). Relative expression of* CD40* mRNA in PBMCs was normalized to the expression level of *β*-actin. All amplification reactions were performed in triplicate.

### 2.6. Western Blot Analysis

Protein was extracted from cultured PBMCs with RIPA Lysis buffer (containing 0.1% PMSF) (Beyotime Biotech, China) following the manufacturer's instructions. Protein samples mixed with loading buffer were denatured at 95°C for 5 min and then naturally cooled. A 100 *μ*g sample was loaded in the gel, and electrophoresis (60 V integrated voltage, 120 V constant voltage) was performed. The semidry transfer method was used to transfer proteins from the gel to polyvinylidene difluoride (PVDF) membranes (Pell, USA) for 48 min. Nonfat milk (5%) was used to block membranes for 1 h. Then, the membranes were immunoblotted with antibodies against CD40 (human monoclonal antibody, Sigma-Aldrich, USA) or *β*-actin (Abcam, UK) followed by a horseradish peroxidase-conjugated secondary antibody. After washing steps, immunoreactive bands were detected by a Bio-Rad Calibrated Densitometer.

### 2.7. Statistical Analysis

Statistical analyses were performed with SPSS 16.0. Significance was based on contingency tables and calculated using a *χ*^2^ test. Multigroup comparisons of the means were carried out by one-way analysis of variance (ANOVA) test with post hoc contrasts by Student–Newman–Keuls (SNK) test. All descriptive results of continuous variables are expressed as the mean ± SD. *P* values less than 0.05 were considered significant.

## 3. Results

### 3.1. Demographic Characteristics of Sepsis Patients and Healthy Controls

The demographic characteristics of both sepsis patients and healthy controls are shown in Table 1. No differences in age, gender, body mass index (BMI), systolic blood pressure (SBP), diastolic blood pressure (DBP), or history of smoking and drinking were found between the two groups.

### 3.2. Distribution of rs1883832 Polymorphism Frequency in Sepsis Patients and Healthy Controls

*CD40* gene sequencing identified three genotypes (CC, CT, and TT) as reported by others [18]. As shown in Table 2, the frequency of the TT genotype was significantly higher in the sepsis patient group compared with the healthy control group (20.3% versus 17.7%, *χ*^2^ = 11.574, *P* = 0.0031), as was the proportion of rs1883832 T allele carriers (46.9% versus 39.0%, *χ*^2^ = 7.473, *P* = 0.0063, OR = 1.385, 95% CI: 1.096–1.749).

### 3.3. Differences in CD40 mRNA and Protein Expression between Sepsis Patients and Healthy Controls

**Figure 1:**
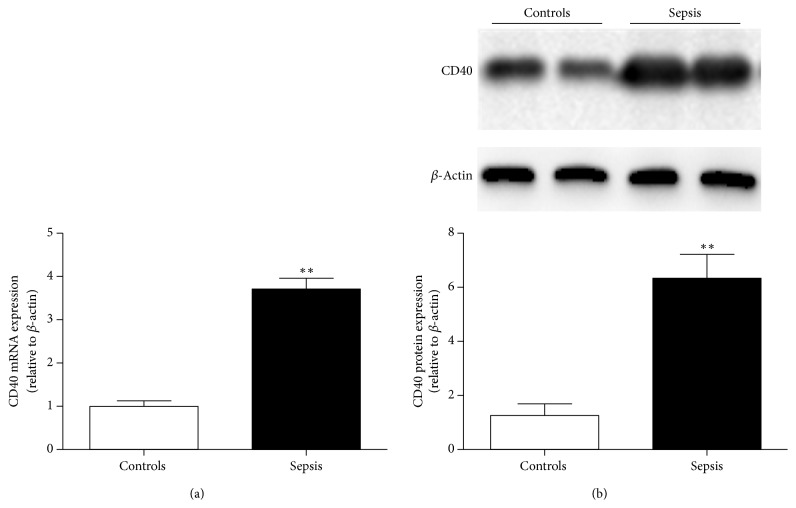
CD40 expression in healthy controls and sepsis patients. (a) CD40 mRNA expression in healthy controls (*n* = 120) and sepsis patients (*n* = 120). (b) Representative immunoblot showing CD40 protein expression in healthy controls (*n* = 80) and sepsis patients (*n* = 80). CD40 expression was significantly higher in sepsis patients compared to healthy controls both at mRNA and protein levels. All data are presented as the mean ± SD. ^*∗∗*^*P* < 0.01 compared with healthy controls.

CD40 plays an important role in many inflammatory diseases including sepsis. Therefore, we isolated PBMCs from both sepsis patients and healthy controls to determine CD40 expression levels. Our data revealed that CD40 expression was significantly higher in sepsis patients compared to healthy controls at both mRNA and protein levels (Figures 1(a) and 1(b)).

### 3.4. Differences in sCD40L Levels between Sepsis Patients and Healthy Controls

**Figure 2:**
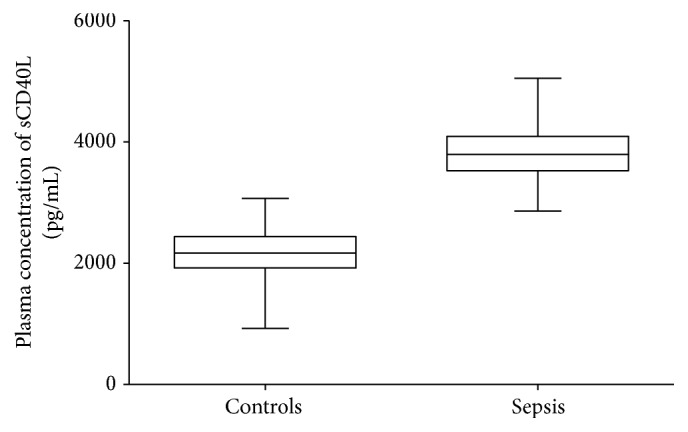
Plasma sCD40L levels in healthy controls and sepsis patients. ELISA was used to determine the plasma sCD40L levels in healthy controls (*n* = 180) and sepsis patients (*n* = 180). Sepsis patients displayed significantly higher sCD40L levels than healthy controls. All data are presented as the mean ± SD.

In this study, we used a sCD40L ELISA kit to measure the plasma sCD40L levels in different groups. We found that sepsis patients displayed significantly higher sCD40L levels than healthy controls (Figure 2).

### 3.5. Association of the CD40 rs1883832 Polymorphism with CD40 Expression

**Figure 3:**
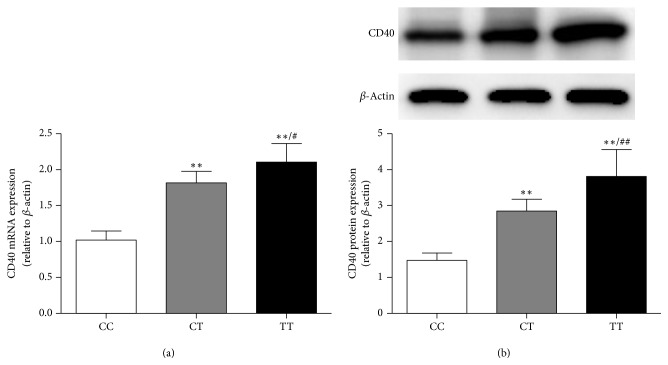
Influence of the CD40 rs1883832 polymorphism on CD40 expression in sepsis patients. (a) Association between CD40 mRNA expression and the CD40 rs1883832 polymorphism in sepsis patients, *n* = 48 per group. (b) Representative immunoblot showing CD40 protein expression by genotype among sepsis patients, *n* = 40 per group. Individuals with the TT genotype displayed the highest mRNA and protein expression of CD40. All data are presented as the mean ± SD. ^*∗∗*^*P* < 0.01 compared with CC genotype carriers. ^#^*P* < 0.05, ^##^*P* < 0.01 compared with CT genotype carriers.

The differences in CD40 expression according to* CD40* genotype were analyzed in PBMCs from both sepsis patients and healthy controls. Our results indicated that the* CD40* rs1883832 T allele carriers among sepsis patients exhibited a significant increase in* CD40* mRNA expression. Among the three genotypes, individuals with the TT genotype displayed the highest mRNA expression of* CD40* (Figure 3(a)). The association of CD40 protein expression with rs1883832 polymorphism was consistent with the* CD40* mRNA expression, and individuals with the rs1883832 T allele showed significantly higher CD40 protein expression than C allele carriers (Figure 3(b)). No significant differences were observed between the* CD40* rs1883832 polymorphism and CD40 expression in healthy controls.

### 3.6. Influence of the CD40 rs1883832 Polymorphism on Plasma sCD40L Concentration

**Figure 4:**
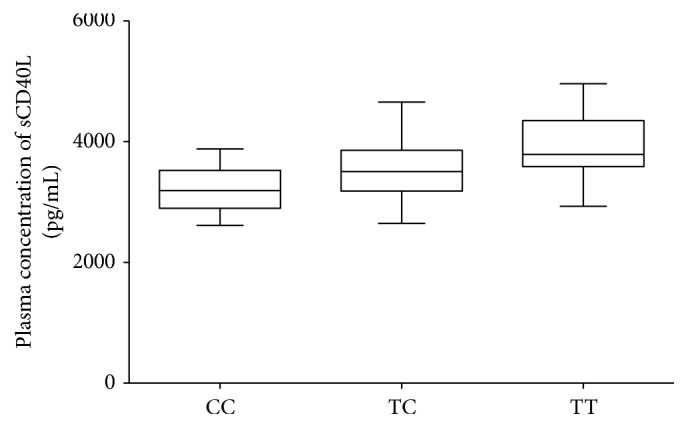
Influence of the CD40 rs1883832 polymorphism on plasma sCD40L levels in sepsis patients. Association between sCD40L level and the CD40 rs1883832 polymorphism in sepsis patients.* CD40* rs1883832 TT carriers exhibited significantly higher plasma sCD40L levels than CT or CC genotype carriers among the sepsis patients. All data are expressed as the mean ± SD, *n* = 48 per group.

Differences in sCD40L levels among* CD40* genotypes were also further investigated in sepsis patients. As shown in Figure 4,* CD40* rs1883832 TT carriers exhibited significantly higher plasma sCD40L levels than CT or CC genotype carriers among the sepsis patients, whereas healthy controls who carried the corresponding polymorphic alleles displayed no significant difference (data not shown). This result is consistent with previous studies, supporting the hypothesis that the* CD40* rs1883832 polymorphism is a functional SNP which may be involved in the process of sepsis through regulating plasma sCD40L levels.

## 4. Discussion

In this research, we first performed a case-control study to explore the potential relationship between the* CD40* rs1883832 polymorphism and sepsis susceptibility. We found that the T allele of* CD40* rs1883832 polymorphism was associated with increased risk of sepsis. In the further study, we investigated the effect of the rs1883832 polymorphism on CD40 expression in PBMCs and on sCD40L levels in plasma. Our results suggested that T allele carriers showed significantly higher CD40 expression (both mRNA and protein levels) and sCD40L levels than C allele homozygotes in sepsis patients.

CD40 is a type I transmembrane receptor protein belonging to the TNF receptor superfamily. The gene encoding* CD40* is located on chromosome 20 (q12-q13.2), and CD40 is expressed on a variety of cell types, including immune cells, endothelial cells, fibroblasts, smooth muscle cells, and platelets. After binding to CD40L, CD40 is activated and internalized into the cell. The activated CD40 then binds to members of the tumor necrosis factor receptor associated factor family (TRAF) and stimulates the NF-kappa B signaling pathway. Finally, proinflammatory genes and procoagulant genes are induced under the regulation of NF-kappa B signaling. Therefore, the CD40/CD40L system serves as a critical link among immune response, inflammation, and a hypercoagulable state in multiple diseases [22–26]. Sepsis is an acute inflammatory disease characterized by SIRS, so our findings that the levels of CD40 and sCD40L are increased in sepsis patients compared with healthy controls support the presence of a systemic inflammatory response state associated with sepsis, as well as being consistent with previous research by others. All these observations make* CD40* an interesting candidate gene for a role in human sepsis.

The rs1883832 polymorphism exists in the Kozak sequence of the* CD40* gene; any SNP in this sequence would lead to abnormal translation efficiency in any related proteins. It is reported that, in a Chinese population, the rs1883832 C/T polymorphism was associated with susceptibility to systemic lupus erythematosus (SLE), and individuals carrying the* CD40* rs1883832 T variant allele tend to have increased sCD40L levels compared to the homozygous wild-type genotype in SLE patients [27]. Zhang et al. also found that the T allele of the rs1883832 polymorphism was associated with ischemic stroke and poststroke epilepsy [18, 20]. Similar associations of the rs1883832 polymorphism were also reported for other diseases including osteoporosis, multiple sclerosis, and Crohn's disease [28, 29]. All these observations suggest a pathogenic role for the rs1883832 T allele. However, some contradictory results have also been reported. Tian and colleagues reported that the -1C allele of the rs1883832 polymorphism was a risk factor that might determine an individual's susceptibility to acute coronary syndrome (ACS) in a Chinese population [30]. Wang et al. also found that the frequency of the C allele in ACS patients was significantly higher than in controls [31]. Similar associations of the C allele with diseases were also found in other studies [32, 33]. Our research concerning the effect of the rs1883832 polymorphism on susceptibility to sepsis gives more reliable data to analyze the function of this SNP. Our results demonstrated that the T allele, not the C allele, was associated with an increased risk of sepsis.

Because of the regulatory function of the rs1883832 polymorphism on the expression of CD40, we isolated mRNA and protein from PBMCs in all subjects to investigate the effect of the rs1883832 polymorphism on CD40 expression. We found that individuals with the T allele showed higher expression of CD40 at both mRNA and protein levels in sepsis patients. In line with our findings, Zhang et al. also have observed that TT genotypes were associated with elevated CD40 expression [20, 27]. However, many other studies also demonstrated that the rs1883832 C allele was associated with increased CD40 expression [31, 33–37]. There are several possibilities that might lead to this difference. First, we should consider the association of the* CD40* rs1883832 polymorphism with CD40 expression in the whole system. Different diseases induce different internal environments in patients. For example, IL-6, TNF-*α*, and HMGB1 levels are significantly higher in sepsis patients than in patients with other diseases or healthy people. These inflammatory factors can also directly or indirectly induce an increase in CD40 expression. Second, even small variations in the* CD40* Kozak sequence can have significant posttranslational effects on CD40 translation. However, other posttranslational modifications should also be considered. For example, the altered miRNA patterns induced by inflammatory states in sepsis patients may influence CD40 expression at both mRNA and protein levels. To our knowledge, Guo et al. have already identified that the* CD40* mRNA is a direct target of miR-145, which is also an inflammation-associated miRNA. In addition, many RNA-binding proteins (HuR, CUGBP1, TIAR, etc.) can recognize the 3′-untranslated region, AU-rich elements, or GU-rich elements in certain mRNAs and regulate their stabilities and translations. The anomalous expressions of these RBPs were also observed in many diseases, which may also induce an increase in CD40 expression. Third, the* CD40* rs1883832 polymorphism may be in linkage disequilibrium with other functional SNPs, such as the upstream rs6074022 SNP in the* CD40* promoter. Therefore, the increased* CD40* mRNA expression may be caused by these SNPs in promoter region.

As described above, the activation of the CD40/CD40L system leads to appreciable upregulations of many proinflammatory genes, which induce high levels of proinflammatory cytokines and oxidative stress. The elevated levels of cytokines and oxidative stress will then stimulate platelets to release a large amount of sCD40L [38]. The platelets are generally considered to be the main source of sCD40L in plasma. In the present study, our data showed that the plasma sCD40L levels were significantly higher in T allele carriers among sepsis patients.

## 5. Conclusion

Based on our findings, we conclude that the rs1883832 polymorphism in* CD40* gene and the levels of sCD40L were significantly associated with the risk of sepsis in a Chinese population. Our study will contribute to identifying a genetic marker that may be used to predict susceptibility to sepsis. When we encounter a sepsis patient with CD40 rs1883832 T allele, it is important to control the sCD40L and other inflammatory parameters into a lower level than C allele carriers to alleviate the sepsis severity. However, the sample size in our experiment was limited and different income social strata of the population was also not considered, so further studies with larger cohorts of sepsis patients and multicenter trials should be performed to explore the correlation of the rs1883832 polymorphism in the* CD40* gene with sepsis susceptibility. Because the expression of CD40 is also regulated by other posttranslational control mechanisms, such as miRNAs and DNA methylation, further research into the underlying regulatory mechanisms of CD40 in sepsis is also needed. Some other markers such as CD80 and CD86 in antigen presenting cells could be potentially correlated to a predictive outcome of the septic patient by increasing the CD40L levels, so these associated markers and several interesting clinically relevant parameters should also be investigated in further research.

## Figures and Tables

**Table 1:** General characteristics of the sepsis patients and healthy controls.

Parameter	Sepsis (*n* = 261)	Controls (*n* = 322)	*P*
Gender (male/female)	175/86	208/114	NS
Age, year	56.45 ± 10.23	57.36 ± 11.66	NS
BMI, kg/m^2^	23.51 ± 2.72	23.85 ± 1.95	NS
SBP, mmHg	128 ± 14	122 ± 18	NS
DBP, mmHg	80 ± 6	84 ± 10	NS
History of smoking	95	106	NS
History of drinking	102	124	NS
APACHE II score	22.3 ± 4.2	N.A	
SOFA score	8.1 ± 1.5	N.A	
Sepsis status			
Sepsis, *n* (%)	57 (21.8)	N.A	
Septic shock, *n* (%)	64 (24.5)	N.A	
Severe sepsis, *n* (%)	140 (53.7)	N.A	
Pathogens, *n* (%)			
Gram-positive	45 (17.2)	N.A	
Gram-negative	85 (32.6)	N.A	
Mixed Gram-positive and Gram-negative	79 (30.3)	N.A	
Fungus	36 (13.8)	N.A	
Negative blood cultures	16 (6.1)	N.A	
Source of infection			
Lung	141 (54.0)	N.A	
Blood	24 (9.2)	N.A	
Abdomen	55 (21.1)	N.A	
Urinary tract	14 (5.4)	N.A	
Catheter-related	7 (2.7)	N.A	
Trauma	14 (5.4)	N.A	
Others	6 (2.3)	N.A	
28-day mortality, *n* (%)	93 (35.6)	N.A	

**Table 2:** The frequencies of different rs1883832 genotypes in sepsis patients and healthy controls.

	*n*	Genotype (%)			Allele (%)	
		CC	CT	TT	C	T
Sepsis patients	261	69 (26.4)	139 (53.3)	53 (20.3)	277 (53.1)	245 (46.9)
Controls	322	128 (39.8)	137 (42.5)	57 (17.7)	393 (61.0)	251 (39.0)
*χ* ^2^			11.574		7.473	
*P* value			0.0031		0.0063	
OR (95% CI)					1.385 (1.096~1.749)	
Hardy-Weinberg			*P*_case_ = 0.264		*P*_control_ = 0.058	
